# COVID-19 Knowledge/Practices, Mental Status, and Return-To-Work Concerns Among Healthcare Workers in Huangzhou District, Huanggang City, China

**DOI:** 10.3389/fpubh.2021.723118

**Published:** 2022-01-14

**Authors:** Yanfei Zheng, Tianxing Li, Ying Zhang, Hui Luo, Minghua Bai, Zhenquan Liu, Yong Zeng, Guangrui Deng, Huiqun Xiao, Yang Zhang, Zheng Zhang, Huirong Chen, Jiangming He, Boyi Li, Yingshuai Li, Ji Wang, Lingru Li, Qi Wang

**Affiliations:** ^1^National Institute of Traditional Chinese Medicine Constitution and Preventive Treatment of Diseases, Beijing University of Chinese Medicine, Beijing, China; ^2^College of Chinese Medcine, Beijing University of Chinese Medcine, Beijing, China; ^3^China Tibetology Research Center, Institute for Tibetan Medicine, Beijing, China; ^4^Health Committee of Huanggang, Huanggang, China; ^5^Huangzhou District People's Hospital, Huanggang, China; ^6^Huangzhou Maternity and Child Health Care Hospital, Huanggang, China; ^7^Huanggang Hospital of Traditional Chinese Medicine (TCM), Huanggang, China; ^8^Huangzhou General Hospital of Huanggang, Huanggang, China

**Keywords:** COVID-19, return to work, healthcare worker, anxiety, traditional Chinese medicine

## Abstract

**Objective:**

This study investigated the COVID-19-prevention knowledge and practices of healthcare workers (HCWs), their psychological states concerning the return to work, and their trust and requirements in using traditional Chinese medicine (TCM) to prevent and treat COVID-19. It is hoped that the study can serve as a reference for policy making during the resumption of work in other countries or regions experiencing similar situations.

**Methods:**

This study comprised a quantitative cross-sectional online survey design. Purposive sampling and Cluster sampling were used to recruit all HCWs working in public hospitals in Huangzhou District, Huanggang City, Hubei Province, China. From April 23 to May 14, 2020, surveys were sent electronically to all 13 public hospitals in this area.

**Results:**

In total, 2,079 responses were received and 2,050 completed forms were included. After analysis, 47.9 and 46.6% of HCWs indicated that they possessed very good knowledge or good knowledge of preventative measures, respectively. Multivariable log-binomial regression indicated that male, tertiary hospital, medical staff, and undergraduate/postgraduate qualification were associated with good knowledge. Good knowledge was also well-correlated with good practice (OR: 3.277; 95% CI: 2.734–3.928; *P* < 0.01). 59.8% of HCWs reported worries about resuming work; especially asymptomatic infections. The Self-Rating Anxiety Scale (SAS) indicated that 10.8% of participants had mild anxiety, 1.5% moderate anxiety, and 0.1% severe anxiety. Female, divorced/widowed, and working in a high risk hospital (the Huangzhou District People's Hospital was used for throat swab examinations of returning workers) were risk factors for concerns about resuming work and anxiety symptoms. However, good preventive knowledge was a protective factor for anxiety. HCWs' trust in using TCM to treat COVID-19 was significantly higher than their trust in using TCM for prevention (*P* < 0.001). Regarding preferences for preventative TCM products, oral TCM granules were the most preferred (62.4%). HCWs also indicated they wanted to know more about the clinical efficacy, applicable population, and adverse reactions of preventative TCM products (89.3, 81.1, and 81.4%, respectively).

**Conclusion:**

While HCWs had good knowledge of COVID-19 preventative measures, this did not eliminate the psychological impact of resumption of work. Promotion of COVID-19 prevention knowledge reduces the risk of infection, and alleviates the worries and anxiety symptoms of HCWs about resuming work (especially in administrative staff, those with low education, and those working in primary hospitals). Additional psychological support is required for female HCWs, divorced/widowed HCWs, and those working in high-risk hospitals. Finally, systematic trials of preventative TCM products are recommended.

## Introduction

Following the outbreak of novel coronavirus disease (COVID-19) in Wuhan, China, in December 2019, COVID-19 spread rapidly around the world and was declared a pandemic by the WHO on March 11, 2020 (1, 2). Over the ensuing months, COVID-19 caused severe damage to human life and to the global economy. As of August 11, 2021, there were 16,550,892 confirmed cases and 4,321,451 deaths. Traditional public health measures achieved good results in China (3), and many provinces were quickly able to contain the number of new confirmed cases. Once the epidemic is under control, economic recovery will be a major agenda, and the resumption of work and production will be a key factor in this process. However, the resumption of work will inevitably increase the risk of infection, with the increased flow of people escalating exposure.

Health care workers (HCWs) have played a key role in the control of COVID-19, and they suffer both tremendous risk of infection and psychological pressure. As of February 11, 2020, front-line medical staff accounted for 3.8% of all confirmed cases, of which 63% were from Wuhan, and more than 70% of the population are estimated to have experienced psychological distress (2, 4, 5). Thus, it is important that HCWs understand and practice preventive measures for COVID-19 and maintain their mental health. With society beginning to resume work and production, throat swab testing of batches of returning workers, and the resumption of medical activities, HCWs are facing uncertain risks, including asymptomatic infected individuals and unknown transmission routes. In this context, we conducted an investigation to learn about HCWs' COVID-19 prevention knowledge and practices, and HCWs' psychological state regarding their return to work. In addition, we investigated their concerns and requirements around returning to normal work practices, with the goals of establishing the problems that HCWs are most concerned about and the conditions that HCWs need, and thus hopefully provide more targeted policies to ensure an orderly return to work.

Traditional Chinese Medicine (TCM) incorporates thousands of years' experience in the prevention and treatment of pandemic and endemic diseases. In China, over 85% of patients with COVID-19 have received TCM treatment on top of conventional therapies (6). This combination of TCM and Western medicine (WM) has been established to be superior to WM alone in improving symptoms, shortening the course of disease and the length of hospital stay, reducing moderate-severe conversion rate, and attenuating side effects associated with conventional therapeutics (7–9). TCM prevention and treatment remedies have been included in China's official diagnosis and treatment guidelines for COVID-19 from the third to the eighth version (10). Based on historical records and direct evidence of SARS and H1N1 influenza prevention, TCM could be an alternative approach for the prevention of COVID-19 in high-risk populations (11). While we were carrying out this study, the COVID-19 vaccine was being developed, and had not yet been applied. In addition to routine prevention methods, such as mask protection and traditional disinfection, TCM has been demonstrated to be one of the most unique and promising prevention methods in China. Hence, we also investigated the attitudes of HCWs to the prevention and treatment of COVID-19 with TCM, and their demand for and preferred form of COVID-19 related TCM prevention products. After determining these needs, we will explore the feasibility of promoting Chinese medicine as a preventive measure for COVID-19. The overall aim of our study is to understand the readiness of HCWs for future resumption of work and production, and to provide a reference for formulating prevention strategies to apply once work and production have resumed.

## Methods

### Study Design and Population

**Figure 1:**
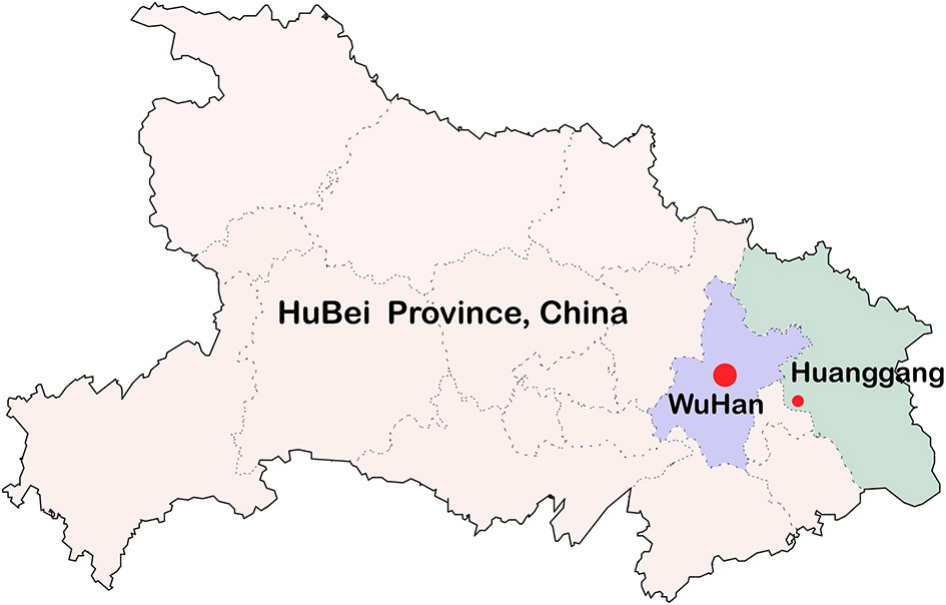
Geographical location of Huanggang, where the respondents were located.

This study was conducted using a quantitative cross-sectional online survey research design. Purposive sampling was used to recruit HCWs working in public hospitals in Huangzhou District, Huanggang City, Hubei Province, China. Huanggang is a prefecture-level city located in the eastern part of Hubei Province, adjacent to Wuhan, which was one of the cities hardest hit by COVID-19 (Figure 1). Huangzhou District is the political and economic center of Huanggang City. The 13 public hospitals under Huanggang City jurisdiction (acting in consortium) played a major role in the fight against the epidemic. On March 30, 2020, the Huanggang municipal government issued a notice of resumption of work and production, and HCWs on secondment started returning to their units to resume normal medical work. Over the following 2 months, each unit in the area arranged for their HCWs to gradually return to work after undergoing a throat swab test (according to unit conditions). At this point, cluster sampling was used to recruit all HCWs in all public hospitals to avoid sampling errors.

### Questionnaire Design

The questionnaire consisted of five main components: demographic characteristics; knowledge and practice of preventative COVID-19 measures; concerns about resuming work; anxiety symptoms; and trust of and demand for TCM-based COVID-19 prevention.

Baseline demographic characteristics such as gender, age, education, marital status, occupation, and work unit were recorded. A self-made questionnaire was used to assess participants' knowledge and practice of preventative COVID-19 measures. Concerning the knowledge part, respondents were asked about their familiarity with preventative measures, and responses were recorded on a scale ranging from 1 (“very familiar”) to 5 (“unfamiliar”). Using the question “What do you know about basic preventive measures against COVID-19?,” participants were provided with 10 options to assess their knowledge of preventive measures. To assess preventative practices, respondents were asked “Do you practice these preventive measures in daily life?,” and responses were recorded on a scale ranging from 1 (“absolutely practice”) to 5 (“Practice none at all”).

For the question assessing whether participants were concerned about resuming work, participants could answer “Yes” or “No.” “Yes” respondents could subsequently choose possible reasons from a list of options provided. Anxiety was assessed using the Self-Rating Anxiety Scale (SAS) (12, 13), which is a validated screening instrument for use among adults. The SAS is a self-reported 20-item scale that asks participants to rate their level of distress for each component during the past seven days. Scores are calculated by summing the scores for each item and multiplying by 1.25 to get the final score. The scores are graded as follows to assess the severity of psychological impact: normal (<50); mild (50–59); moderate (60–69); and severe (>70).

In the final component, the questions were designed to assess participants' trust in using TCM against COVID-19, whether they believed TCM preventative methods are needed to deal with the risks of resuming work, what types of methods they prefer, and what information about TCM preventative measures they would like to receive. TCM constitution, guided by TCM theories, classifies humans into nine constitution types according to physiological and psychological features and adaptive capacity, i.e., balanced constitution, qi-deficiency constitution, yang-deficiency constitution, yin-deficiency constitution, phlegm-dampness constitution, damp-heat constitution, blood stasis constitution, qi stagnation constitution, and an inherited special constitution. The TCMC results were obtained using the adult edition of the Rating Scale for 9 TCM Constitutions which has 60 evaluation items for a self-rating scale (14, 15) (**Additional File 3**).

### Data Collection

From April 23 to May 14, 2020, we distributed the electronic questionnaires to all 13 public hospitals in Huangzhou District, Huanggang City, including one tertiary hospital, five secondary hospitals, and seven primary hospitals. Huangzhou District People's Hospital had the additional responsibility of providing throat swab tests to people returning to work in Huanggang (providing more than 800 examinations a day, and reaching more than 1,200 during peak periods). Because of this added burden, Huangzhou District People's Hospital was considered separately as a high-risk hospital. HCWs in all hospitals were grouped into occupational hierarchies based on the risk of COVID-19. The two main groups were medical staff (doctors and nurses) and administrative staff (including logistics workers). Complete questionnaires concluded from April 23 to May 14, 2020 were considered qualified and were included in the analysis. Incomplete questionnaires and participants who had been quarantined due to diagnosed COVID-19 or a positive swab test were excluded. All respondents provided informed consent.

### Statistical Analysis

SPSS 21.0 was used for data analysis (SPSS, Inc., Chicago, IL, USA). Significance was set at α = 0.05, and all tests were two-tailed. Qualitative variables are described by frequency distribution. A two-tailed chi-squared test was used to compare the distribution of nominal data. The non-parametric Mann–Whitney *U*-test and Kruskal–Wallis test were used to compare ordinal data between two or more groups. We analyzed the relationships between participants' COVID-19 knowledge, their symptoms of anxiety and worry, and their characteristics using log-binomial models to estimate relative risk (RR) and corresponding 95% confidence intervals (CIs). The response variables were all binary categorical variables. A five-level Likert scale questionnaire (good to bad) was used to subjectively evaluate participants' knowledge and practice of COVID-19 preventive measures. The responses were then classified as “Good knowledge” or “Common knowledge” using the option settings and the capacity of the results for division. Anxiety and worry were also transformed into binary variables, with 1 denoting yes and 0 denoting no. The lower value binary variable was chosen as the reference category.

## Results

### Demographic Characteristics

For this cross-sectional survey, we retrieved a total of 2,079 questionnaires from 13 hospitals. After 29 questionnaires were excluded as incomplete, 2,050 questionnaires were used in our analyses, giving an overall response rate of 94.6%. Most participants were female (1557, 76.0%) in the 31–40 years old age range. Most had completed junior college or above (1758, 85.8%), and 68.6% were unmarried. A total of 550 participants worked at Huangzhou District General Hospital (high risk of infection), and 1,500 worked at the other hospitals (lower risk of infection). The majority were medical staff (1,941, 94.7%), while 109 were administrative staff (5.3%). Secondary hospitals accounted for the highest proportion of participants (1,095, 53.4%), followed by tertiary (602, 29.4%), and primary hospitals (353, 17.2%) (Table 1).

**Table 1:** Baseline characteristics of study participants (*N* = 2,050).

**Variables**	***N***	**%**
**Gender**		
Male	493	24.0
Female	1,557	76.0
**Age (years)**		
<30	823	40.1
31–40	550	26.8
41–50	488	23.8
>50	189	9.2
**Education**		
High school and below	292	14.2
College	754	36.8
Bachelor's	965	47.1
Master's	39	1.9
**Marital status**		
Single	557	27.2
Married	1,407	68.6
Other^b^	86	4.2
**Risk level of hospital**		
Huangzhou district people's hospital	550	26.8
Others^a^	1,500	73.2
**Job category**		
Medical staff	1,941	94.7
Administrative staff	109	5.3
**Hospital grade**		
Tertiary	602	29.4
Secondary	1,095	53.4
Primary	353	17.2

a *other hospitals;*

b *divorced or widowed*.

### Knowledge and Practice of Preventative COVID-19 Measures

Overall, 47.9% of the participants chose “very familiar” and 46.6% chose “relatively familiar.” Since the capacities of the next three levels were small (5.3, 0.2, and 0%), the lower three levels were merged with Level 2 into one category, yielding a “Good knowledge” category (Level 1) and a “Common knowledge” category (Levels 2–5). On chi-squared tests, the variables significantly related with knowledge levels were gender (χ^2^ = 20.797, *P* < 0.001), job category (χ^2^ = 14.255, *P* < 0.001), hospital grade (χ^2^ = 13.598, *P* = 0.001), and education level (χ^2^ = 33.244, *P* = <0.001). Age, different risk levels of hospital, and marital status were not statistically significantly related with self-reported knowledge level (Table 2).

**Table 2:** Association between HCWs' COVID-19 knowledge^a^ and their characteristics.

**Variables**	**Good knowledge *N* (%)**	**Common knowledge *N* (%)**	**χ^2^**	***p***
**Gender**			20.797	<0.001^**^
Male	280 (56.8)	213 (43.2)		
Female	701 (45.0)	856 (55.0)		
**Job category**			14.255	<0.001^**^
Medical staff	948 (48.8)	993 (51.2)		
Administrative staff	33 (30.3)	76 (69.7)		
**Risk level of hospital**				
Huangzhou district people's hospital	281 (51.1)	269 (48.9)	3.157	0.076
Others^b^	700 (46.7)	800 (53.3)		
**Hospital grade**			13.598	0.001^**^
Tertiary	310 (51.5)	292 (48.5)		
Secondary	532 (48.6)	563 (51.4)		
Primary	139 (39.4)	214 (60.6)		
**Age (years)**			2.388	0.496
<30	382 (46.4)	441 (53.6)		
31–40	278 (50.5)	272 (49.5)		
41–50	230 (47.1)	258 (52.9)		
>50	91 (48.1)	98 (51.9)		
**Education**			33.244	<0.001^**^
High school or below	115 (39.4)	177 (60.6)		
College	323 (42.8)	431 (57.2)		
Bachelor's	518 (53.7)	447 (46.3)		
Master's	25 (64.1)	14 (35.9)		
**Marital status**			1.710	0.425
Single	255 (45.8)	302 (54.2)		
Married	687 (48.8)	720 (51.2)		
Other^c^	39 (45.3)	47 (54.7)		

** *p < 0.01;*

a *Knowledge based on participants' self-evaluation; “very familiar” was considered “good knowledge”; the rest were considered “Common knowledge.”*

b *12 other public hospitals.*

c *Divorced or widowed*.

With knowledge status as the dependent variable (good knowledge = 1, common knowledge = 0), a log-binomial model and multivariable regressions were performed to identify the main factors affecting HCWs' knowledge. In the adjusted analyses, tertiary hospital (RR: 1.216; 95% CI: 1.043–1.416; *P* = 0.012), male gender (RR: 1.242; 95% CI: 1.128–1.368; *P* < 0.001), medical staff (RR: 1.568; 95% CI: 1.173–2.095; *P* = 0.002), and undergraduate/postgraduate qualification were associated with good knowledge (Table 3).

**Table 3:** Association between participant characteristics and COVID-19 prevention knowledge.

	**RR**	**95% CI**	***P*-value**
**Gender**			
Male	1.242	1.128–1.368	<0.001^**^
Female	1		
**Job category**			
Medical staff	1.568	1.173–2.095	0.002^**^
Administrative staff	1		
**Unit**			
Low-risk hospitals	1.035	0.914–1.173	0.589
High-risk hospital	1		
**Hospital grade**			
Tertiary	1.216	1.043–1.416	0.012^*^
Secondary	1.142	0.976–1.336	0.097
Primary	1		
**Age (years)**			
<30	0.895	0.738–1.086	0.260
31–40	0.903	0.755–1.079	0.259
41–50	0.908	0.761–1.084	1.084
>50			
**Education**			
High school or below	0.687	0.516–0.913	0.010^*^
College	0.772	0.599–0.995	0.046^*^
Bachelor's	0.916	0.721–1.163	0.472
Master's	1		
**Marital status**			
Single	0.961	0.741–1.245	0.761
Married	1.011	0.803–1.272	0.927
Other^a^	1		

** *p < 0.01;*

* *p < 0.05; Log-binomial model adjusted for all independent variables listed in the table simultaneously.*

a *divorced or widowed*.

Among the preventive measures, wearing masks was known to everyone, followed by washing hands frequently (2033, 99.2%), and reducing the numbers of people gathering (1958, 95.5%) (**Additional File 2**; Supplementary Table 1). Participants' information sources were mainly official channels such as TV news, official online platforms, and government notices (97.2%) (**Additional File 2**; Supplementary Table 2).

Regarding practices, the survey assessed the degree of implementation of preventive measures in daily life. Overall, 46.5% chose “absolutely practice,” and 49.3% chose “practice more” (the other three levels accounted for 3.8, 0.1, and 0.3%). Using the analogy knowledge classification method, we divided practice into two categories (combining the last three levels into Level 2). We then conducted a chi-square test between knowledge and practice. The results reveal that the higher the HCWs' knowledge of COVID-19 prevention, the more measures they practiced (OR: 3.277; 95% CI: 2.734–3.928; *P* < 0.01) (Table 4).

**Table 4:** Cross-table of knowledge and practice of COVID-19 prevention measures (*N* = 2,305).

		**Practice**			**OR (95% CI)**	***P*-value**
		**Good**	**Common**	**Total**		
Knowledge					3.277 (2.734–3.928)	<0.001^**^
Good	*N*	603	378	981		
	Row %	61.5%	38.5%	100.0%		
	Column %	63.3%	34.5%	47.9%		
Common	*N*	350	719	1069		
	Row %	32.7%	67.3%	100.0%		
	Column %	36.7%	65.5%	52.1%		
Total	*N*	953	1,097	2,050		
	Row %	46.5%	53.5%	100.0%		
	Column %	100.0%	100.0%	100.0%		

** *p < 0.01; Pearson's χ2 = 169.700, p = 0.000*.

### Psychological Status Concerning Resuming Work and the Influencing Factors

Overall, 59.8% (1,226) of participants reported worries about resuming work. Asymptomatic infection was the main cause of concern (89.2%, 1,093), followed closely by gathering of employees in the workplace (84.6%, 1,037), contact with people during commutes (74.5%, 913), and insufficiently disinfected workplaces (74.1%, 908) (**Additional File 2**; Supplementary Table 3). A total of 855 of the HCWs had children returning to school, and 91.6% were worried about the consequences. HCWs were mainly concerned about students gathering in closed, small classroom spaces (96.8%, 758), followed by concerns about children's hygiene practices (e.g., washing hands, wearing masks) (88.4%, 692) (**Additional File 2**; Supplementary Table 4). Compared to their own concerns about resuming work, HCWs were more worried about their children returning to school (χ^2^ = 115.647, *P* < 0.01).

**Figure 2:**
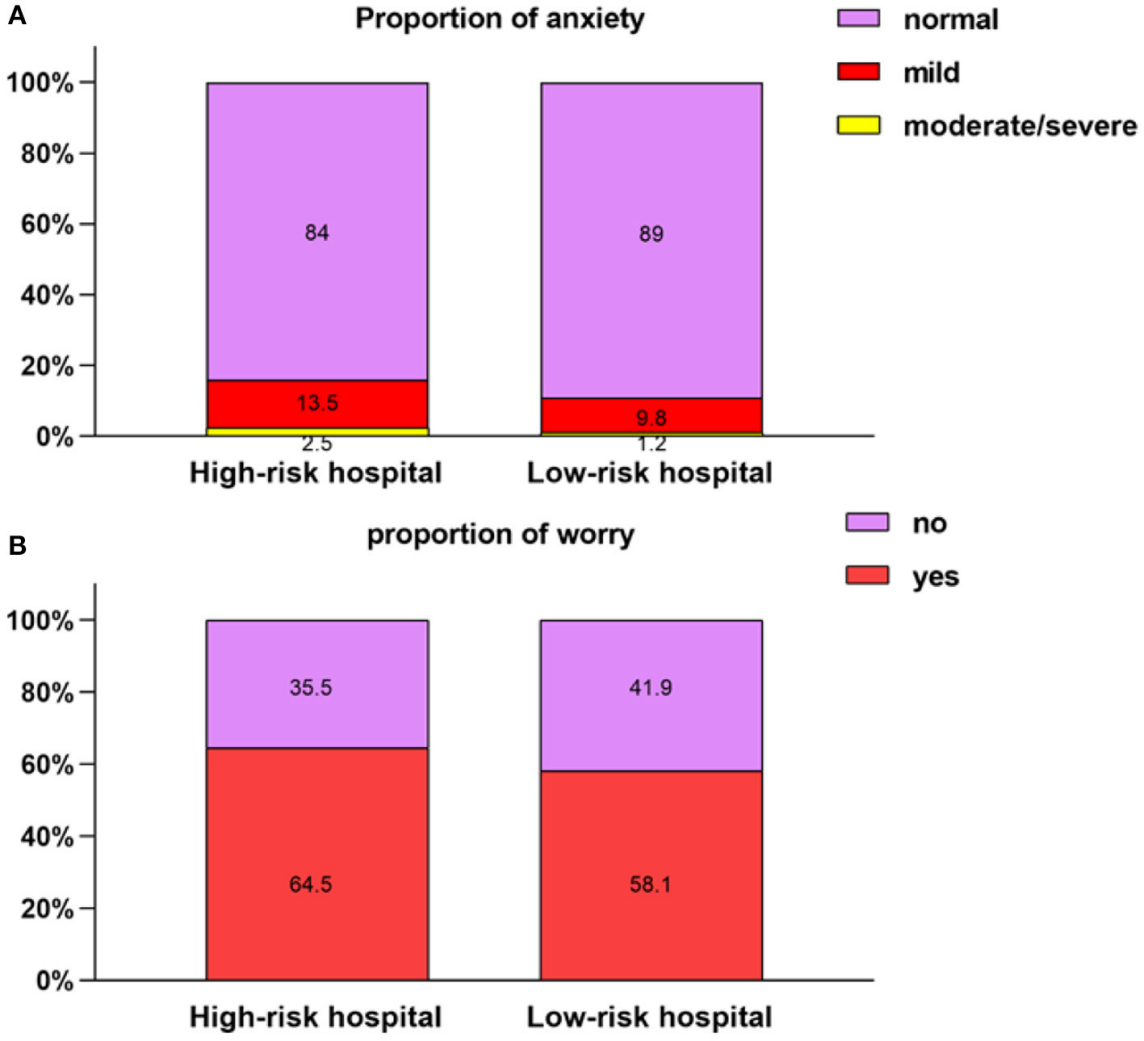
Comparison of psychological features between two groups. **(A)** Proportion of anxiety. **(B)** Proportion of worry.

In addition, we used the SAS scale to investigate HCWs' anxiety states, 10.8% of HCWs had mild anxiety, 1.5% moderate anxiety, and 0.1% severe anxiety. Next, we analyzed the factors that might have influenced worry and anxiety among the participants (Table 5). The adjusted results indicate that females were more likely to have anxiety and worry. Marital status was also an influencing factor on mental status. Compared with divorced/widowed HCWs, single HCWs (RR = 0.573, 95% CI: 0.388–0.845) had a lower risk of worry. As a designated throat swab screening hospital for returning to work, the People's Hospital of Huangzhou District bears a higher infection risk. This factor affected two psychological characteristics of the participants. As shown in Figure 2 and Table 6, HCWs working at a high-risk hospital (Huangzhou District People's Hospital) had higher anxiety levels than HCWs working at low-risk hospitals (16.1 vs. 10.0%, respectively). Among HCWs at the high-risk hospital, 13.5% had mild anxiety, and 2.5% moderate to severe anxiety; the corresponding proportions were 9.8 and 1.2%, respectively, for those at low-risk hospitals. The differences in the severity of anxiety between the two groups were significant (*P* = 0.002). Furthermore, 64.5% of HCWs at the high-risk hospital reported worries about resuming work, while only 58.1% of HCWs at low-risk hospitals expressed such worries; again, the differences between the two groups were significant (*P* = 0.008). Knowledge of preventive measures was also an influencing factor of mental state. Good knowledge reduced the risk of anxiety (RR: 1.385; 95% CI: 1.116–1.718).

**Table 5:** Multivariable analysis of anxiety and worry based on different characteristics.

**Variables**	**Anxiety**		**Worry**	
	**RR (95% CI)**	***p***	**RR (95% CI)**	***p***
**Gender**				
Male	0.643(0.464–0.893)	0.008**	0.810 (0.736–0.891)	<0.001^**^
Female	1		1	
**Job category**				
Medical staff	1.658 (0.867–3.170)	0.127	0.914 (0.795–1.051)	0.206
Administrative staff	1		1	
**Unit**				
High-risk hospital	1.614 (1.172–2.223)	0.003^**^	1.162 (1.060–1.274)	0.001^**^
Low-risk hospitals	1		1	
**Knowledge**				
Common knowledge	1.385 (1.116–1.718)	0.003^**^	1.071 (0.999–1.149)	0.053
Good knowledge	1		1	
**Hospital grade**				
Tertiary	1.110 (0.809–1.523)	0.520	1.048 (0.941–1.167)	0.398
Secondary	0.722 (0.499–1.046)	0.085	0.985 (0.882–1.100)	0.782
Primary	1			
**Marital status**				
Single	0.674 (0.413–1.100)	0.115	0.769 (0.644–0.918)	0.004^**^
Married	0.784 (0.497–1.237)	0.295	1.006 (0.859–1.178)	0.943
Others	1			
**Constitution**				
Qi-deficiency	7.057 (5.309–9.381)	<0.001^**^	1.137 (0.976–1.326)	0.100
Yang-deficiency	4.599 (3.247–6.514)	<0.001^**^	1.285 (1.133–1.458)	<0.001^**^
Yin-deficiency	6.067 (4.084–9.012)	<0.001^**^	1.037 (0.815–1.319)	0.767
Phlegm-dampness	4.348 (2.398–7.882)	<0.001^**^	0.856 (0.584–1.254)	0.425
Damp-heat	1.776 (.288–10.951)	0.536	0.980 (0.491–1.955)	0.954
Qi-stagnation	6.445 (4.613–9.005)	<0.001^**^	1.167 (0.971–1.402)	0.100
Blood stasis	3.591 (2.412–5.347)	<0.001^**^	1.053 (0.906–1.224)	0.501
Inherited special	2.788 (1.460–5.324)	0.002^**^	0.788 (0.572–1.084)	0.143
Balanced	1			

** *p < 0.01; Log-binomial model adjusted for all independent variables listed in the table simultaneously*.

**Table 6:** Different severity levels of anxiety and worry among the participants.

**Variables**	**High-risk hospital** ***N* = 550**	**Low-risk hospitals** ***N* = 1,500**	***N***	***p***
**Anxiety**				0.002^a^^**^
Normal	462 (84.0)	1335 (89.0)	1797 (87.7)	
Mild	74 (13.5)	147 (9.8)	221 (10.8)	
Moderate	13 (2.4)	17 (1.1)	30 (1.5)	
Severe/extreme	1 (0.2)	1 (0.1)	2 (0.1)	
**Worried**				0.008^b^^**^
Yes	355 (64.5)	871 (58.1)	1226 (59.8)	
No	195 (35.5)	629 (41.9)	824 (40.2)	

** *p < 0.01;*

a *p-value for two independent-sample Mann–Whitney tests;*

b *p-value for chi-squared test*.

TCMC is a unique concept in TCM. TCMC provides a systematic method that includes physiology and psychology to divide people into nine different types. In the sense of modern medicine, a balanced constitution is a healthy person, while the other eight constitutions, representing unbalanced states of Qi, blood, Yin, Yang, phlegm, or dampness (the viewpoint of TCM philosophy), are similar to the sub-healthy state of modern medicine (9). According to TCM theories, disease occurrence is due to an imbalance in the body caused by external factors. To address this imbalance, corresponding intervention methods are adopted for different physical types to restore body balance and help prevent diseases. Our results indicate that those with an unbalanced constitution were more likely to have anxiety (except in the case of damp-heat constitution). Several imbalanced constitutions, including qi-deficiency (RR: 7.057; 95% CI: 5.309–9.381), qi-stagnation (RR: 6.445; 95% CI: 4.613–9.005), and yin-deficiency (RR: 6.067; 95% CI: 4.084–9.012), posed higher risks for anxiety than a balanced constitution.

### HCWs' Requirements for Resuming Work and Attitudes Regarding TCM COVID-19 Prevention

We next investigated HCWs' requirements for resuming work. Thorough workplace disinfection and the provision of adequate protective tools (e.g., masks, alcohol) had the highest positive response rates (92.7 and 93.3%, respectively). Taking temperature measurements as people enter the workplace was also high on HCWs' requirements (85.6%). Canceling activities that require crowd gathering and reviewing whether staff meet the requirements for resuming work yielded 82.8 and 79.7% positive response rates, respectively. Receiving training on COVID-19 prevention was a requirement of 75.1% of HCWs, while 65.7% indicated a need for preventive products. Among all requirements for resuming work, providing dedicated buses had the lowest positive response rate (40.7%) (**Additional File 2**; Supplementary Table 5). Regarding students returning to school, the abovementioned items accounted for a higher proportion (**Additional File 2**; Supplementary Table 6).

**Figure 3:**
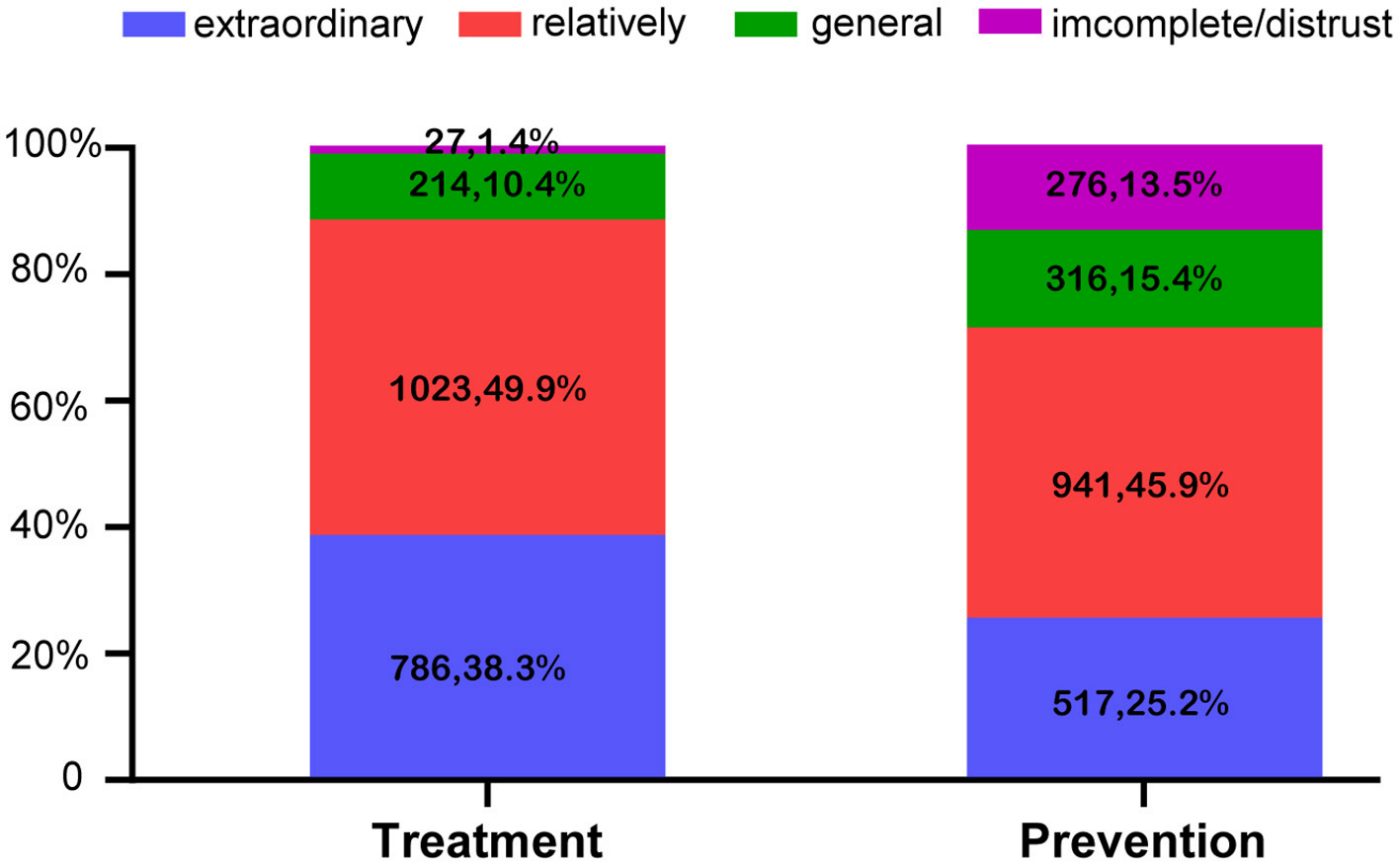
Trust in using TCM to prevent and treat COVID-19 (*N* = 2,050).

Finally, we investigated HCWs' trust in using TCM to treat and prevent COVID-19. Figure 3 shows that trust in using TCM to treat COVID-19 was higher than trust in using it for prevention (*P* < 0.001, Wilcoxon signed-rank test); 38.4% believed TCM could effectively treat COVID-19, while 25.2% believed TCM could prevent COVID-19. Overall, only 1.3% expressed little or no trust in the ability of TCM to treat COVID-19. In comparison, 13.5% had little or no trust in the ability of TCM to prevent COVID-19. We next investigated HCWs' TCM dosage preferences, and the information they would like to receive about preventative TCM products. As to the preferred form of TCM, 62.4% of HCWs chose oral granules, 27.2% chose external sachets, and 9.2% chose topical sprays. Among the 44 (1.2%) participants who chose other forms, 44% preferred oral capsule/tablet/pill formulations. Most HCWs (89.3%, 1,743) expressed an interest in receiving information about the clinical efficacy of preventative TCM products. Moreover, 81.1% (1584) wanted information about the applicable population for the products, and 81.4% (1,588) wanted information about possible adverse reactions. Product-development information ranked the lowest (46.7%, 905). HCWs were also asked to choose their level of demand for TCM products (low, moderate, high, very high), with the proviso that the preventive products for COVID-19 were developed by an authority. The results for very high, high, moderate, and low demand were 23.3, 29.2, 41.2, and 6.4%, respectively.

## Discussion

This study was conducted at a time when the epidemic was under control and society was facing the resumption of work and production. Our study focused on HCWs' knowledge and practice of COVID-19 preventive measures, their psychological status, and their concerns and requirements for resuming work. Our aim is to inform training and policies for people returning to work and to guide HCWs in prioritizing protection and avoiding occupational exposure. Our study also provides a basis for developing strategies to improve the mental health of HCWs, thus relieving concerns and ensuring an orderly and efficient return to work. This analysis of HCWs' knowledge and the factors affecting their practices should provide a reference for formulating targeted prevention and public policies for HCWs, especially in high-risk areas like Huanggang. Of the HCWs surveyed in this study, 94.5% were familiar with COVID-19 prevention procedures, although medical staff demonstrated a higher knowledge level than administrative staff. Moreover, HCWs from tertiary hospitals and HCWs with undergraduate/postgraduate qualifications also showed higher knowledge levels. Our results are in line with multiple previous findings. For example, Xu et al. and Saqlain et al. reported that 89 and 93.2% of participants, respectively, have sufficient knowledge regarding COVID-19 (16, 17). A number of factors are known to influence COVID-19 prevention knowledge, including gender, age, education, and occupation, etc. (18). While previous studies have reported that gender and good knowledge are not clearly correlated (19, 20), male participants in our study demonstrated higher levels of knowledge.

Knowledge is a prerequisite for establishing prevention understanding and promoting positive behaviors, and an individuals' cognition toward disease affects their behaviors to a certain extent (21). Our investigation revealed a positive correlation between knowledge and practice of preventive measures, which is consistent with previous research. Thus, a good understanding of preventive measures is the main premise for promoting practice. Our results indicate that health education measures should target groups of different cultural levels, occupations, and genders to improve their prevention knowledge and their practice of preventive measures. These classes could be conducted through hospital organized training, as 23 interviewees specifically mentioned. HCWs' information sources were mainly official channels (97.2%) and community notifications (89.9%), followed by portals/news clients (65.4%), and online social tools (52.5%). Although health authorities have been consistently disseminating COVID-19 information since the disease was first detected, there has also been a surge in false and inaccurate information (22). This overload of information is likely to have caused confusion and difficulties in ascertaining the correct information. In this regard, official institutions can make use of social media tools to provide more targeted and authoritative information about COVID-19, thus combating the spread of unreliable information.

Overall, 59.8% of HCWs were concerned about resuming work, and 12.4% of HCWs suffered anxiety symptoms (including moderate to extremely-severe anxiety symptoms in 1.6% of HCWs). Our results suggest that the psychological states of HCWs is a problem that needs attention and guidance. Asymptomatic infection (89.2%) and crowd gathering (84.6%) were the main causes of concerns, consistent with the frequent occurrence of asymptomatic infections at the time (23). In the early and peak stages of the COVID-19 outbreak, HCWs faced enormous psychological pressures, and many demonstrated symptoms of anxiety, depression, insomnia, and Burnout Syndrome; more than 70% were reported to experience psychological distress (2, 3, 24). Compared to these figures, our cohort reported lower prevalence rates of anxiety, especially moderate to severe anxiety (2, 25–28). Thus, although reduced, psychological pressures concerning the resumption of work during the pandemic remained. Being female and working in a high-risk hospital were risk factors for concerns about resuming work and anxiety symptoms. Some studies have reported a higher prevalence of mental health problems among women during the COVID-19 outbreak. For example, women have reported a higher fear of COVID-19 beyond infection (29), and a higher likelihood of anxiety/depression/PTSD symptoms (30).

HCWs in close contact with COVID-19 patients (and at a higher risk of infection) also experience more psychological disorders, exhibiting almost twice the risk (compared to non-clinical staff) of suffering anxiety and depression (31). These reports are consistent with our own results. Compared to HCWs at the other hospitals, HCWs at Huangzhou District People's Hospital reported more severe anxiety and worry. As noted above, Huangzhou District People's Hospital screened a large number of people for throat swab tests to assist their return to work. Thus, HCWs at this hospital faced a greater flow of people, and higher exposure to infectious patients (32). The reasons for more severe anxiety may be related to “hypochondriac concerns” (i.e., excessive worry about being infected), and fear that the epidemic was hard to control (33). In terms of job attributes, one previous study investigated differences in the psychological status of medical staff and administrative staff during the COVID-19 pandemic, with medical staff reporting greater fear, anxiety, and depression than administrative staff (31). Medical staff may also suffer from higher risk of anxiety (compared to administrative staff) during the period of work resumption. In general, the pressures experienced by medical staff have been greater. (1) Medical staff have shouldered and continue to shoulder the majority of the work of epidemic prevention in hospitals. In particular, medical staff have been responsible for screening and collecting nasopharyngeal swabs and have been in close contact with patients' secretions, resulting in higher exposure risks and greater psychological pressures. (2) Medical staff are also subjected to increased work intensities and work stresses (compared to administrative staff). In our present study, medical and administrative staff did not exhibit significant differences in their mental health. This could be related to the large difference in sample size between the two groups—there were only 109 administrative staff. In addition, family factors also affected the psychological states of the interviewees, with divorced/widowed HCWs at higher risk of concern. This outcome is also consistent with the results of a large number of studies. Isolation during difficult periods may affect mental health, and HCWs with high work stresses undoubtedly benefit from family support at this time (34).

During the COVID-19 outbreak, the government issued a number of policy documents addressing the mental and physical health needs of HCWs, including provision of a place for adequate rest with food and supplies, and proper provision of protective equipment (35, 36). We devised the present study, because we believed it was similarly important to help HCWs reduce their concerns about returning to work and to reduce their anxiety symptoms after the peak of the COVID-19 epidemic. We propose that all HCWs, but especially females, divorced/widowed, and those working in high-risk hospitals, receive adequate support, including psychological counseling, long-term mental health management, and flexible policies for work resumption.

In our survey of HCWs, we found that good knowledge of COVID-19 prevention measures reduced anxiety symptoms. This is consistent with previous research indicating that useful and trustworthy information encouraged perceptions that the outbreak could be controlled by protective behaviors and lowered the prevalence of depression and anxiety (20). Positive preventive measures such as the frequent practice of hand hygiene, the wearing of face masks, and improvements in workplace hygiene could decrease the likelihood that people would experience stress, anxiety, depression, and insomnia (37). These findings further highlight the need for the promotion of the knowledge and practice of preventive measures.

Because of a lack of specific antiviral therapeutics and vaccines for COVID-19, conventional medicines (including broad-spectrum antibiotics, antivirals, and corticosteroids) and TCM are both used for the treatment of COVID-19 patients in China. The combination of TCM and Western medicine (WM) was superior to WM alone in improving symptoms, shortening the course of disease and the length of hospital stay, reducing moderate-severe conversion rate, and attenuating side effects associated with conventional therapeutics (7–9). Furthermore, TCM has a long history of epidemic prevention (11), and has achieved relatively good results for SARS (a related coronavirus that causes pneumonia) (38, 39). TCM prevention and treatment remedies have been included China's official diagnosis and treatment guidelines for COVID-19 from the third to the eighth version (10). However, clinical evidence for the use of TCM to prevent COVID-19 is generally inadequate. A single arm study revealed good preventive effects for TCM decoctions on close contacts of patients with COVID-19 (38). A large prospective cohort study using proprietary Chinese medicines provided evidence that TCM intervention could reduce the occurrence of flu symptoms, although neither the test group nor the control group included confirmed cases of COVID-19 (40). In our survey, 77.1% of HCWs held a positive attitude toward TCM prevention of COVID-19 (15.4% remained neutral and 13.5% held a negative attitude). Overall, HCWs reported a high degree of trust in TCM prevention, which is consistent with a previous study conducted among medical professionals in Sichuan, China (41). Likewise, Chinese immigrants to Canada also reported their belief that TCM was an effective means of preventing COVID-19, and they reported that they would use it if they were experiencing COVID-19 symptoms (42).

Concerning their requirement for preventive TCM products developed by an authority, 93.7% of HCWs indicated such a need (with 52.5% indicating a large need). These results remind us that TCM can be used to supplement conventional COVID-19 prevention methods to facilitate a resumption of work and a return to normal life. Even after an efficient vaccine has been made available, TCM remains a very worthy prevention method to choose (taking into account the medical burden). HCWs did express the need for a focus on safety and efficacy in preventive TCMs. Thus, attention should be paid to the evaluation of the safety and superiority of TCM, and to the disclosure of this data. Concerning TCM delivery, 62.4% preferred oral granules; this is related to the perception that TCM involves drinking decoction. Moreover, 88.2% of HCWs expressed a more positive attitude toward TCM treatment of COVID-19 compared with TCM prevention (77.1%). Ostensibly, this may be because TCM prevention lags behind TCM treatment in terms of popularization and publicity. However, the good clinical efficacy of TCM for the treatment of COVID-19 may be a more critical reason. Although most provinces in China have issued preventive programs recommending Chinese herbal formulae (11), TCM is not routinely used in COVID-19 prevention. To address this shortfall, pharmacokinetic studies should be conducted to obtain the pharmacokinetic profile of TCM products, including their absorption, distribution, metabolism, and excretion parameters. Clinical trials should then be conducted to test their preventive efficacy and safety on human subjects.

Finally, we also investigated the constitution type of HCWs. Constitution is a TCM concept that focuses on individual differences in people, of which there are nine basic constitution types (43). People with different constitutions may have different psychological characteristics (44). Previous studies have found that those with a “phlegm-dampness” constitution and a “qi-deficiency” constitution suffer more from the effects of COVID-19 (45). Our studies reveal that an imbalanced constitution increased the risk of anxiety (especially with qi-deficiency, qi-stagnation, and yin-deficiency constitutions). A dysfunction in the hypothalamic-pituitary-adrenal (HPA) axis could be related to the connection between constitution and anxiety (46, 47). Therefore, HCWs' TCMC types could be used as a reference for the development of COVID-19 related products.

This study has several limitations. First, and foremost, we used a cross-sectional research method to assess COVID-19 knowledge/practices and mental status during the return to work, and work and production have now largely been resumed. In our study, we reveal the important needs and psychology of HCWs during the process of resumption of work and production. The provision of policies addressing these needs is a very important measure performed by the Chinese government, which has thus far managed the resumption of work and production to great effect. TCM has played an important role in COVID-19 prevention and control during this period, and this study is of great value in this context. Second, the self-evaluation method and absence of objective test questions in the survey may have influenced HCWs' knowledge. Third, the SAS was used to investigate anxiety symptoms, and the information provided on symptoms was not verified by a medical professional. The data analysis parameter using “worry” as a psychological symptom may have been unreliable, since the only options were “yes” or “no.” Fourth, the questionnaire did not subdivide medical staff, such as doctors and nurses, by department category. Lastly, the study was limited in scope. Societies that experienced the COVID-19 outbreak and are facing the resumption of work are represented here by Huanggang. Although sporadic cases sometimes occur, the epidemic in China has been mostly contained. However, it may be assumed that our research remains meaningful, because of the constant threat of COVID-19 throughout the globe.

## Conclusion

HCWs from public hospitals in Huanggang demonstrated a good knowledge of COVID-19 preventative measures during the period covering the resumption of work after the epidemic was contained. This was especially true for male HCWs, HCWs in tertiary hospitals, medical staff, and HCWs with a higher degree. However, good knowledge of COVID-19 preventative measures did not completely eliminate the psychological impact of work resumption. Thus, 59.8% of HCWs reported worries about resuming work, and 12.4% reported anxiety symptoms. Supporting HCWs is an important public health measure for ensuring an orderly and efficient return to work, and in helping to prevent the further spread of COVID-19. It is especially important that public health measures include measures to avoid infection among HCWs and to support their mental health. Special training on COVID-19 prevention measures is recommended to reduce the risk of infection and to relieve mental problems. The provision of adequate protective equipment and the thorough disinfection of the workplace are other vital measures. In addition, humanistic care and psychological counseling should be provided to HCWs, in particular female HCWs, divorced/widowed HCWs, HCWs with insufficient knowledge, and HCWs in high-risk hospitals. Although TCM demonstrates potential for COVID-19 protection, there is currently a lack of reliable evidence. To address this, preventative TCM products should be developed further, and *in vivo* and *in vitro* trials should be conducted. Only when reliable evidence is provided, can TCM be confidently used to supplement conventional COVID-19 prevention methods. It is hoped that this study can serve as a reference for policy making during the resumption of work in countries or regions facing similar situations.

## Data Availability Statement

The datasets presented in this study can be found in online repositories. The names of the repository/repositories and accession number(s) can be found below: This can be found at https://doi.org/10.6084/m9.figshare.15087891.v1.

## Ethics Statement

The studies involving human participants were reviewed and approved by Beijing University of Chinese Medicine. The patients/participants provided their written informed consent to participate in this study.

## Author Contributions

LL and JW: study design and data collection. TL: data analysis and writing. MB, YanfZ, GD, HX, YiZ, ZZ, HC, and JH: data collection. YoZ, HL, and BL: data analysis guidance. YangZ, LL, ZL, and QW: study design and give direction to the paper. All authors contributed to the article and approved the submitted version.

## Funding

This work was supported by the Key Research and development projects of the Ministry of Science and Technology (2020YFC0845200).

## Conflict of Interest

The authors declare that the research was conducted in the absence of any commercial or financial relationships that could be construed as a potential conflict of interest.

## Publisher's Note

All claims expressed in this article are solely those of the authors and do not necessarily represent those of their affiliated organizations, or those of the publisher, the editors and the reviewers. Any product that may be evaluated in this article, or claim that may be made by its manufacturer, is not guaranteed or endorsed by the publisher.
