# Supplementary material for: COVID-19 Knowledge/Practices, Mental Status, and Return-To-Work Concerns Among Healthcare Workers in Huangzhou District, Huanggang City, China
Source: Front Public Health. 2022 Jan 14;9:723118. doi: 10.3389/fpubh.2021.723118 (PMC8795632; doi:10.3389/fpubh.2021.723118)
Supplement: Supplementary file 3 [file Data_Sheet_2.PDF]

No. □□□□□□

Body Constitution in Traditional Chinese Medicine Questionnaire, Designed by Wang Qi

Beijing University of Chinese Medicine
Constitution and Reproductive Medicine Research Center
June 2013

Survey Consent Form and Right-to-Know Information

Dear Interviewee,

You are invited to join an important doctorate study designed by the Body Constitution and Reproductive Medicine Research Center , Beijing University on the topic: ***TCM Body Constitution of the General Public and Relevant Factors***

We appreciate your participation. The survey is free and your answers are anonymous to public.

Please take a moment to carefully read the following information in its entirety; understand the aim, significance and procedures of the study; and provide your consent.

Study Overview and Aim
Based on the nine-constitution theory in traditional Chinese medicine, the aim of study is to examine and observe the constitution of the general public; investigate the influence of nationalities, regions, living habits and other objective conditions on the types of constitution; analyze the difference in TCM body constitution features among people in different social status; and further explore those factors causing such differences with the goal of developing preventive and healthy strategies for different groups of people based on the types of body constitution.

Description of the Survey Process
The study will objectively assess your current TCM body constitution and general health. Should you decide to participate, we will inquire about your general situation, past history of illnesses, current health situation, and type of constitution by this questionnaire.

Benefits and Risks
Benefits: Participants will receive a personalized health care analysis and recommendations.
Risks: We appreciate your willingness to share your valuable time to complete the survey. This survey has no predictable potential risks, and investigators make every effort to eliminate possible issues that may endanger the participants.
Expense and Compensation: This survey will not charge any fee from the interviewees and the Beijing University of Chinese Medicine will not provide any financial rewards in exchange for your participation.

Principle of Voluntary Participation
It is assumed that survey participation is on a completely voluntary basis.

Protection of Privacy
Your survey information will be kept confidential. You can complete this survey anonymously. If you do choose to provide your name or e-mail address for follow-up, your name will not appear in any study report or publications. Your survey data will be stored using a coding system to protect the privacy of every individual interviewee. Moreover, the identity of the interviewees will not be disclosed upon release of the survey report. Throughout the study period, the researchers will exert all efforts to protect the privacy of interviewees.

Liability
The Beijing University of Chinese Medicine and its research affiliates have no policy or plan to pay for any liability claim that may arise as a result of participation in this survey and research reporting.

Follow-up
The researcher would like to follow-up with those participants willing to engage in future surveys a 6 months, 9 months, 1 year and 18 months. If you are willing to receive follow-up surveys please enter your e-mail address. ___________________________

Interviewee Consent
By beginning to complete the survey, I declare that I have read, and understand, the above statements and voluntarily agree to participate in this study. Furthermore, I understand that prior to starting the survey; I have been given the full opportunity to have any additional questions/concerns addressed by contacting telephone number.

Mark of the interviewee____________________ Date:______________

Researcher: Donna Winges, Doctoral Candidate of Traditional Chinese Medicine
Body Constitution and Reproductive Medicine Research Center
Beijing University of Chinese Medicine
Tel: + 86 136 9920 4068 Email: dm.winges@gmail.com

Wang Qi's Body Constitution Classification Questionnaire

This questionnaire aims to survey your constitution and to subsequently provide a reference for future health management and clinical diagnosis. Please read every question carefully and choose the most suitable response based on your actual situation or experience in the past year. If you are unsure of the answer to a specific question, choose the answer that is most similar to your actual situation. Make sure that you answer all the questions based on your situation **in the past year**, (excluding the effect of drugs) and give only one answer to each question.

Explain:【No】Never happened in the past year.【Slightly】Occasionally happened in the past year.

【Sometimes】Sometimes it happened, but no regular pattern. 【Often】It happened most of the time in the past year.

【All the time】It happened all the time in the past year.

**Experience/condition in the past year**	**No**	**Slightly**	**Sometimes**	**Often**	**All the time**
(1) Were you energetic?	1	2	3	4	5
(2) Did you get tired easily?	1	2	3	4	5
(3) Did you experience shortness of breath?	1	2	3	4	5
(4) Did you get palpitations?	1	2	3	4	5
(5) Did you get dizzy easily or become dizzy when standing up?	1	2	3	4	5
(6) Did you prefer quietness and not like to talk?	1	2	3	4	5
(7) Was your voice weak when talking?	1	2	3	4	5
(8) Did you feel in low spirits and depressed?	1	2	3	4	5
(9) Did you easily feel anxious and worried?	1	2	3	4	5
(10) Did you feel overly sensitive, vulnerable or emotionally upset?	1	2	3	4	5
(11) Were you easily scared or frightened?	1	2	3	4	5
(12) Did you experience distention in the underarm or breast?	1	2	3	4	5
(13) Did you feel chest or abdominal stuffiness?	1	2	3	4	5
(14) Did you sigh without reason?	1	2	3	4	5
(15) Did your body feel heavy or lethargic?	1	2	3	4	5
(16) Did the palms of your hands or soles of your feet feel hot ?	1	2	3	4	5
(17) Did your hands or feet feel cold or clammy?	1	2	3	4	5
(18) Did you feel cold easily in your abdomen, back, lower back or knees?	1	2	3	4	5
(19) Were you sensitive to cold and tended to wear more clothes than others?	1	2	3	4	5
(20) Did your body and face feel hot?	1	2	3	4	5
(21) Did you feel more vulnerable to the cold than others (winter coldness, air conditioners, fans, etc.)?	1	2	3	4	5
(22) Did you catch colds more easily than others?	1	2	3	4	5
(23) Did you sneeze even when you *did not* have a cold?	1	2	3	4	5
(24) Did you have a runny or stuffy nose even when you *did not* have a cold?	1	2	3	4	5
(25) Did you cough due to seasonal changes, temperature changes, or unpleasant odors?	1	2	3	4	5
(26) Did you sweat easily when your physical activity increased slightly?	1	2	3	4	5
(27) Did you forget things easily?	1	2	3	4	5
(28) Did you have an excessively oily forehead and/or T-zone?	1	2	3	4	5
(29) Were your lips redder than in the past?	1	2	3	4	5
(30) Did you have allergies? (e.g. medicine, food, odors, pollen, pet dander, or during seasonal or weather change etc.)	1	2	3	4	5

**Experience/condition in the past year**	**No**	**Slightly**	**Sometimes**	**Often**	**All the time**
(31) Did you get hives/urticaria easily?	1	2	3	4	5
(32) Did your skin have purpura (purple spots, ecchymosis) due to allergies?	1	2	3	4	5
(33) Did black or purple bruises appear on your skin for no reason?	1	2	3	4	5
(34) Did your skin turn red and show traces when you scratched it?	1	2	3	4	5
(35) Did your skin or lips feel dry?	1	2	3	4	5
(36) Did you have visible capillary (thread) veins on your cheeks?	1	2	3	4	5
(37) Did you feel pain somewhere in your body?	1	2	3	4	5
(38) Did you experience hot flashes?	1	2	3	4	5
(39) Did your nose or your face feel greasy, oily, or shiny?	1	2	3	4	5
(40) Did you have a dark face or get brown spots easily?	1	2	3	4	5
(41) Did you get acne or sores easily?	1	2	3	4	5
(42) Did you have upper eyelid swelling?	1	2	3	4	5
(43) Did you get dark circles under the eyes easily?	1	2	3	4	5
(44) Did your eyes feel dry and you used eye drops?	1	2	3	4	5
(45) Were your lips darker, more blue or purple than usual?	1	2	3	4	5
(46) Did you often feel parched and need to drink water?	1	2	3	4	5
(47) Did your throat feel strange (i.e., as if something was stuck or there was a lump in your throat)?	1	2	3	4	5
(48) Did you have a bitter or strange taste in your mouth?	1	2	3	4	5
(49) Did your mouth feel sticky?	1	2	3	4	5
(50) Was your abdomen flabby?	1	2	3	4	5
(51) Did you have an abundance of phlegm, especially in your throat?	1	2	3	4	5
(52) Did you feel uncomfortable when you drank or ate something cold, or did you avoid to drinking or eating cold items?	1	2	3	4	5
(53) Could you adapt yourself to external natural or social environment changes?	1	2	3	4	5
(54) Did you easily experience insomnia?	1	2	3	4	5
(55) Did you easily contract diarrhea when you were exposed to cold or ate (or drank) something cold?	1	2	3	4	5
(56) Did you pass sticky stools and/or feel that your bowel movement was incomplete?	1	2	3	4	5
(57) Did you get constipated easily or have dry stools?	1	2	3	4	5
(58) Did your tongue have a thick coating?	1	2	3	4	5
(59) Did your urethral canal feel hot when you urinated, or did your urine have a dark color?	1	2	3	4	5
(60) Was your vaginal discharge yellowish (**only for female interviewees**)?	1	2	3	4	5
(60) Was your scrotum always wet (**only for male interviewees**)?	1	2	3	4	5

General Questionnaire

Please tick (✓) the right □ and fill in the proper content on the designated lines.

Gender: □Male □Female	Age:___years old Date/time of birth: ____________
City:____________ State: ______________<br>Zip: ______________	Blood type: □A □B □ AB □ O<br>□Unknown

Nationality: □US □Canada □Europe □Australia and New Zealand □Other__________

Ethnicity (click all that apply): □American Indian/Alaska Native □Black □East Asian
□Latin or Hispanic □Middle Eastern □Native Hawaiian/Other Pacific Islander □South Asian
□White □Other _____________________

Marital Status: □Single □ Married □ Divorced □Separated □Widowed □Living w/partner

Education: □Under high school □High school □ Technical School □University graduate
□Post graduate

Profession (current or previous):
□Managerial occupations □ Professional occupations □Technicians □Clerical, Sales or Service occupations □Agricultural, Fishery, Forestry, or related occupations □Processing /Machine trades occupations □Military □Stay at home parent □Student □Retiree □ Other

Eating and drinking habits and preferences for cigarette, alcohol, and tea:
□Sweet □Spicy □Sour □Salty □Light flavor □Oily □Grilled □Cold □Hot
□Smoking □Alcohol □Tea □Coffee □No special preference □Other

Sleeping habits: □Sleeps early and wakes up early □Sleeps late and wakes up late □Sleeps early and wakes up late □Sleeps late and wakes up early □Irregular sleeping habits □Wakes frequently
□Dreams Average sleeping hours per day: About _______hours

Exercise habits：□ Daily □ Regularly □Sometimes □Seldom

History of allergy: □Yes (□Pollen/Ragweed □Seafood □Season change □Mite/Dust □Nuts □Pets dander □Latex □Others_________) □None

History of disease: □Yes □None
□1.CARDIOVASCULAR/NEUROLOGICAL □ High blood pressure □ Arteriosclerosis, coronary heart disease □ Cerebrovascular disease □ Ischemic stroke □ Epilepsy
□2.PULMONARY □ Chronic bronchitis □ Bronchial asthma □ COPD □ Tuberculosis
□3.DIGESTIVE □ Peptic ulcer □ Hepatic disease (□chronic hepatitis, □cirrhosis, □fatty liver) □ Cholecystitis
□4.METABOLIC □ Diabetes □ High Cholesterol □ Obesity □ Gout
□5.URINARY/REPRODUCTIVE □ Nephritis, chronic nephritis □ Prostatitis /Chronic prostatitis □ Gynecological disease (Vaginitis, etc.) □ Polycystic ovary syndrome □ Menopausal syndrome
□6.SKIN □ Psoriasis □ Eczema □ Hives □Acne
□7.ENDOCRINE □ Thyroid □ Auto immune Disorder
□8.MUSCLE /JOINT □ Lumbar disc herniation □ Arthritis □ Sciatic Pain □ Neck Pain □ Low Back Pain □ Knee Pain
□9.PSYCHOLOGICAL □ Mental disorder □ Depression □ Anxiety □ Other ________
□10.GENERAL □ Allergic disease(Allergic rhinitis, Seasonal Allergy etc) □ Osteoporosis □ Hereditary congenital disease □ Malignant cancer_______________ □ Others_________

Family history (disease of direct family members): □Yes □None
□High blood pressure □Heart disease □Alzheimer's □Diabetes □High Cholesterol□Hyperlipidemia □Obesity □Stroke □Epilepsy □Auto immune Disorder □Hepatic disease (chronic hepatitis, cirrhosis, fatty liver) □Kidney Problems □Hereditary congenital disease □Mental disorder ________________ □Allergic disease □Cancer_____________ □ Others___________________

Height:_______ ft.inches (eg.5.7)	Weight: ________pounds	Waist:__________ inches

Blood pressure (high pressure/low pressure):______________/_____________mmHg

Current Medications:____

Rand 36-Item Short Form Health Survey (developed at RAND as part of the Medical Outcomes Study)

1. In general, would you say your health is:

Excellent   Very good   Good   Fair   Poor

2. **Compared to one year ago**, how would you rate your health in general **now**?

Much better now than one year ago   Somewhat better now than one year ago   About the same   Somewhat worse now than one year ago   Much worse now than one year ago

The following items are about activities you might do during a typical day. Does **your health now limit you** in these activities? If so, how much?

**(Circle One Number on Each Line)**	Yes, Limited a Lot	Yes, Limited a Little	No, Not limited at All
3. **Vigorous activities**, such as running, lifting heavy objects, participating in strenuous sports	1	2	3
4. **Moderate activities**, such as moving a table, pushing a vacuum cleaner, bowling, or playing golf	1	2	3
5. Lifting or carrying groceries	1	2	3
6. Climbing **several** flights of stairs	1	2	3
7. Climbing **one** flight of stairs	1	2	3
8. Bending, kneeling, or stooping	1	2	3
9. Walking **more than a mile**	1	2	3
10. Walking **several blocks**	1	2	3
11. Walking **one block**	1	2	3
12. Bathing or dressing yourself	1	2	3

During the **past 4 weeks**, have you had any of the following problems with your work or other regular daily activities **as a result of your physical health**?

**(Circle One Number on Each Line)**	Yes	No
13. Cut down the amount of time you spent on work or other activities	1	2
14. **Accomplished less** than you would like	1	2
15. Were limited in the **kind** of work or other activities	1	2
16. Had **difficulty** performing the work or other activities (for example, it took extra effort)	1	2

During the **past 4 weeks,** have you had any of the following problems with your work or other regular daily activities **as a result of any emotional problems** (such as feeling depressed or anxious)?

**(Circle One Number on Each Line)**	Yes	No
17. Cut down the **amount of time** you spent on work or other activities	1	2
18. **Accomplished less** than you would like	1	2
19. Didn't do work or other activities as **carefully** as usual	1	2

20. During the **past 4 weeks,** to what extent has your physical health or emotional problems interfered with your normal social activities with family, friends, neighbors, or groups?

(Circle One)

Not at all   Slightly   Moderately   Quite a bit   Extremely

21. How much **bodily** pain have you had during the **past 4 weeks**?
(Circle One)
None Very mild Mild Moderate Severe Very severe

22. During the **past 4 weeks,** how much did **pain** interfere with your normal work (including both work outside the home and housework)?
(Circle One)
Not at all A little bit Moderately Quite a bit Extremely

These questions are about how you feel and how things have been with you **during the past 4 weeks**. For each question, please give the one answer that comes closest to the way you have been feeling.
How much of the time during the **past 4 weeks** . . .

**(Circle One Number on Each Line)**	All of the Time	Most of the Time	A Good Bit of the Time	Some of the Time	A Little of the Time	None of the Time
23. Did you feel full of pep?	1	2	3	4	5	6
24. Have you been a very nervous person?	1	2	3	4	5	6
25. Have you felt so down in the dumps that nothing could cheer you up?	1	2	3	4	5	6
26. Have you felt calm and peaceful?	1	2	3	4	5	6
27. Did you have a lot of energy?	1	2	3	4	5	6
28. Have you felt downhearted and blue?	1	2	3	4	5	6
29. Did you feel worn out?	1	2	3	4	5	6
30. Have you been a happy person?	1	2	3	4	5	6
31. Did you feel tired?	1	2	3	4	5	6

32. During the **past 4 weeks,** how much of the time has your **physical health or emotional problems** interfered with your social activities (like visiting with friends, relatives, etc.)?
(Circle One)
All of the time Most of the time Some of the time A little of the time None of the time

How TRUE or FALSE is <u>each</u> of the following statements for you.

**(Circle One Number on Each Line)**	Definitely True	Mostly True	Don't Know	Mostly False	Definitely False
33. I seem to get sick a little easier than other people	1	2	3	4	5
34. I am as healthy as anybody I know	1	2	3	4	5
35. I expect my health to get worse	1	2	3	4	5
36. My health is excellent	1	2	3	4	5

Thank you for your time in completing this survey!
